# Development of a floxed *Gabbr2* gene allows for widespread conditional disruption of GABBR2 and recapitulates the phenotype of germline Gabbr2 knockout mice

**DOI:** 10.1371/journal.pone.0318760

**Published:** 2025-12-15

**Authors:** Julie Rose Hens, Stacey Brown, Pawel Licznerski, Jacqueline Suarez, Elizabeth Jonas, John J. Wysolmerski

**Affiliations:** Department of Internal Medicine, Endocrinology and Metabolism Section, Yale University, New Haven, Connecticut, United States of America; University of Florida, UNITED STATES OF AMERICA

## Abstract

GABBR1 and GABBR2 are widely expressed in the brain and genetic inhibition of their function leads to neurologic dysfunction and premature death in mice. Given that GABBR1 and GABBR2 heterodimerize to form a functional receptor, global knockout of GABBR1 or GABBR2 results in a similar phenotype, characterized by spontaneous epileptiform activity, hyperlocomotor activity, hyperalgesia, impaired memory and premature death. Both GABBR1 and GABBR2 are expressed in a variety of tissues outside the nervous system and GABA-B receptors have been shown to heterodimerize with other class C GPCRs. However, the neurologic consequences of global GABBR1 or GABBR2 knockout mice have made it difficult to study the effects of loss of GABBR function in other organs. While a conditional knockout for GABBR1 is available, the *Gabbr2* gene had not been “floxed”. Therefore, we used CRISPR to insert loxP sites into the *Gabbr2* locus in mice. These mice are normal at baseline but when bred with mice expressing Cre-recombinase under the control of the ubiquitously expressed Actin gene promoter, Gabbr2^lox/lox^ mice recapitulate the phenotype of global GABBR2 knockout mice demonstrating alterations throughout the brain, including the cortex, hippocampus and cerebellum. We document abnormal neurological function, increased neuronal cell death, changes in neuronal architecture, and premature death. These mice should be useful tools to study cell type-specific loss of GABBR2 function in the brain and other organs.

## Introduction

The GABA-B receptors (GABBR1 and GABBR2) are class C, G-protein-coupled receptors (GPCRs) that heterodimerize to form a receptor complex responding to gamma-aminobutyric acid (gaba), the major inhibitory neurotransmitter in the brain. The GABBR1 subunit contains the gaba-binding site, whereas the GABBR2 subunit is responsible for interacting with G-proteins. Furthermore, GABBR1 contains an endoplasmic reticulum (ER) retention site, which prevents its trafficking to the plasma membrane [1]. However, heterodimerization with GABBR2 allows interactions between the coiled-coil sequences of each subunit, masking the ER retention site in GABBR1, and allowing translocation of the heterodimeric complex to the plasma membrane [2,3]. Most commonly, the heterodimeric receptor couples to G_i_ or G_o_, leading to inhibition of adenylate cyclase activity, inositol triphosphate synthesis, voltage-gated calcium channels, and potassium channels [4,5]. As a result, activation of GABABRs hyperpolarizes neurons and inhibits the release of several neurotransmitters, resulting in the suppression of neuronal activity in many brain areas.

GABBR1 and GABBR2 are widely expressed in the brain and genetic inhibition of their function leads to widespread neurologic dysfunction in mice. Given that GABBR1 and GABBR2 heterodimerize to form a functional receptor, global knockout of either GABBR1 or GABBR2 causes a similar phenotype; characterized by spontaneous epileptiform activity, hyperlocomotor activity, hyperalgesia, impaired memory and premature death [6]. As these results demonstrate, GABA-B receptors clearly have important functions in the brain. However, it is now known that both GABBR1 and GABBR2 are expressed in a variety of tissues outside the nervous system [6–9]. Furthermore, it has been shown that the GABA-B receptors can heterodimerize with other class C GPCRs, for example, the extracellular calcium-sensing receptor (CaSR) [10,11]. Studies *in vitro* have demonstrated that interactions with either GABBR1 or GABBR2 can alter CaSR signaling in human embryonic kidney (HEK) cells and breast cancer cells [8,12]. Furthermore, the CaSR and GABBR1 interact in chondrocytes in the growth plate and in parathyroid cells *in vivo* [7]. In the parathyroid glands, GABBR1 modulates CaSR-mediated PTH secretion and systemic calcium metabolism [13], demonstrating that GABBR’s can modulate signaling from other receptors.

The neurological consequences of global loss of function of GABBR1 or GABBR2 have made it difficult to study the effects of loss of GABBR function in other organs in vivo. While a conditional knockout for GABBR1 is available, the GABBR2 gene had not been “floxed”. Therefore, we have used gene editing techniques to insert loxP sites into the GABBR2 locus in order to produce a mouse model that would enable study of GABBR2 in vivo in organs other than the brain. These mice are normal at baseline but when crossed with mice expressing Cre-recombinase under the control of the ubiquitously expressed Actin gene promoter, they recapitulate the phenotype of global GABBR2 knockout mice. These mice will be useful tools to study cell type-specific loss of GABBR2 function in the brain and other organs.

## Materials and methods

### Generation and breeding of Gabbr2 cKO Mice

**Fig 1:**
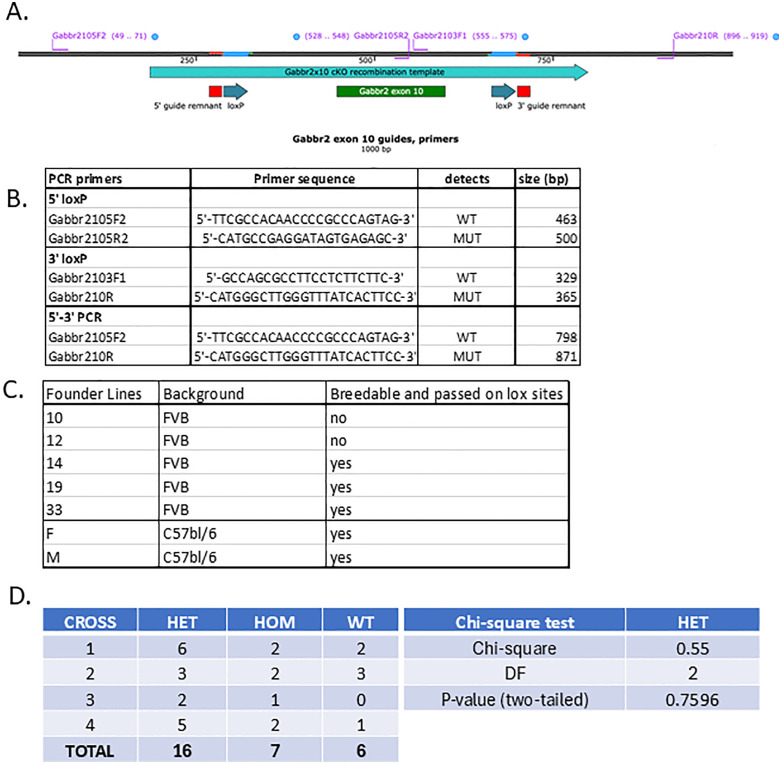
CRISPR design to insert loxP sites into the Gabbr2 gene. A) Map of Gabbr2 gene showing the guide RNAs used to create LoxP sites. B) Primers used to identify loxP sites in Actin-CRE Gabbr2^lox/lox^ mice. C) Table summarizing the different Gabbr2^lox/lox^ mouse lines generated. D) Crossing of heterozygous (Gabbr2^lox/wt^) male and female mice produces offspring with mendelian inheritance pattern. Chi-square test was used to determine whether the observed offspring from lines 19 and 33 were consistent with expected numbers of offspring assuming mendelian inheritance. HET = heterozygous, HOM = homozygous, WT: wild type.

The GABBR2 cKO mouse model was generated via CRISPR-Cas9 genome editing [14–16]. Potential Cas9 target guide (protospacer) sequences in introns 9 and 10 were screened using the online tool CRISPOR http://crispor.tefor.net [17] and candidates were selected. Templates for sgRNA synthesis were generated by PCR, sgRNAs were transcribed *in vitro* and purified (Megashortscript, MegaClear; ThermoFisher). sgRNA/Cas9 RNPs were complexed and tested for activity by zygote electroporation, incubation of embryos to blastocyst stage, and genotype scoring of indel creation at the target sites. The sgRNAs that demonstrated the highest activity were selected for creating the floxed allele. Guide RNA (gRNA) sequences are as follows: intron 9, 5’ guide: ACTAGATCCTCTCACCCAGT and intron 10, 3’ guide CTGCCATGCTGTGACCCCAT. Accordingly, a 615 base long single-stranded DNA (lssDNA) recombination template incorporating the 5’ and 3’ loxP sites was synthesized (IDT) (Fig 1A). C57Bl6 3 SJL F2 or FVB/NJ zygote embryos were transferred to the oviducts of pseudopregnant CD-1 foster females using standard techniques [16,18]. Genotype screening of tissue biopsies from founder pups was performed by PCR amplification and Sanger sequencing to verify the floxed allele. Genotyping of individual animals utilized PCR-based on the primers in Fig 1B. Germline transmission of the correctly targeted allele (i.e., both loxP sites *in cis*) was confirmed by breeding and sequence analysis. Seven potential founders with a floxed Gabbr2 gene were identified, and three (#33, #14, #19) true-breeding FVB lines were generated (Fig 1C). We also generated two true-breeding lines on a C57bl/6 mouse background. The studies described herein were performed on animals derived from lines 33 and 19. The two lines were maintained separately, but because of similar phenotypes, data from the two lines have been pooled except where indicated. Genotyping was performed utilizing a step-wise PCR reaction. PCR began with an initial phase of 8 cycles in which the annealing temperature was increased stepwise by 1 °C per cycle, starting at 65 °C and reaching 72 °C, to gradually enhance primer–template specificity. Each of these cycles included denaturation at 95 °C, annealing at an incrementally increased temperature, and extension at 72 °C. This was followed by 30 standard amplification cycles consisting of denaturation at 95 °C for 30 seconds, annealing at 55 °C for 30 seconds, and extension at 72 °C for 30 seconds. A final extension step at 72 °C for 5 minutes was then performed to complete synthesis of any unfinished products. Inheritance was confirmed to be Mendelian using results from 4 crosses between heterozygous males and females from founder lines 19 and 33 and analyzed with Chi-square analysis (Fig 1D).

We crossed Gabbr2 ^lox/lox^ mice with B6.FVB-*Tmem163*^*Tg(ACTB-cre)2Mrt*^/EmsJ (Actin-cre) to verify the effectiveness of gene excision between the CRISPR-generated lox sites. Actin-Cre/Gabbr2 ^lox/lox^ mice (cKO) express Cre recombinase ubiquitously under the control of the human beta-actin promoter beginning at the blastocyst stage of development, generating a global GABBR2 knockout mouse.

All studies were performed on animals between the ages of 3 and 15 weeks. Specific ages of the mice are described for the individual experiments. Isoflurane anesthesia was used for specific experiments as indicated. Mice were euthanized with either excess CO_2_, or isoflurane overdose. All procedures were approved by the Yale University Animal Care and Use Committee and followed U.S. National Institutes of Health standards.

### RNA and protein analysis

Brains from 3-week-old mice were removed and total RNA was isolated. One µg of RNA was converted to cDNA using Applied Biosystems high-capacity cDNA reverse transcription kit (Thermo Fisher Scientific, Waltham, MA). Taqman probes were used to measure GABBR1(Mm00444578_m1), GABBR2(Mm01352554_m1), and GAPDH(#4352339E) (Thermo Fisher Scientific, Waltham, MA). Real-time qPCR was performed using TaqMan ^TM^ Fast Universal PCR Master Mix reagents (Thermo Fisher Scientific, Waltham, MA) and Applied Biosystems StepOne Plus Real-Time PCR System. Ct values were analyzed using the ΔΔ – Ct method [19].

For protein isolation, half the brain cut in the coronal mid-line was added to 1 ml of RIPA buffer (50 mM Tris HCl pH 8, 150 mM NaCl, 1% NP-40, 0.5% sodium deoxycholate, 0.1% SDS) with complete mini protease inhibitors (Roche Diagnostics, Mannheim, Germany). Using a TissueLyser II (Qiagen, Germantown, MD) with a 5 mm bead, tissue was lysed for 2 minutes at 30 rotations per second. Lysates were incubated on ice for an hour, before being centrifuged at 12,000 g, for 20 minutes. Thirty micrograms of protein were loaded in a well. Samples were not heated, and after the transfer, blots were blocked for 1 hour in 5% milk with 0.1% Tween-20. Primary antibodies were added at 1/1000 overnight at 4 °C while rocking. Blots were washed with PBS, and then goat-anti rabbit or goat-anti mouse secondary antibody was added for an hour and samples were washed in PBS. Blots were imaged using Odyssey Li-Cor system. Results were normalized to actin.

We used the following antibodies to GABBR1 (#ab55051, Abcam, Waltham, MA), GABBR2 (#ab75838, Abcam, Waltham, MA), actin (#MA5-11869, Invitrogen, Rockford Illinois), IRDye® 800CW Goat anti-Mouse IgG (H + L) (Li-Cor, Lincoln, Nebraska) IRDye®, 680RD Goat anti-Rabbit IgG Secondary Antibody (Li-Cor, Lincoln, Nebraska)

### Histology

Mouse brains were examined in animals that were 8–10 weeks old. Brains were embedded in paraffin blocks and 5-micron sections were acquired. Sections were stained with hematoxylin and eosin, and Luxol fast blue [20]. Immunohistochemistry was performed with S100 antibody (Thermo Cat# RB-9018-R7, Thermofisher Waltham, MA) to examine changes in myelination in the central nervous system. In brief, tissue was deparaffinized and rehydrated to distilled water. Following rehydration, the slides were placed in Tris-buffered saline 0.1% Tween-20, which serves as the buffer for all subsequent washing steps. Anti-S100 antibody was then applied at a dilution of 1:4 for 30 minutes. Slides were rinsed in TBS and then incubated with HRP-conjugated secondary immunoglobulin (Mach2 Biocare Medical, Cat# RHRP520, Biocare Medical, Pacheco, CA) per manufacturer’s instructions. The presence of the target antigen was visualized using diaminobenzidine (DAB) chromogen (Cat # BDB2004, Biocare Medical, Pacheco, CA) per manufacturer’s instructions. Slides were counterstained with hematoxylin, dehydrated, cleared, and mounted with TissueTek Glass mounting media (Cat # NC2606967, Fisher Scientific, Suwanee, GA) for microscopic examination.

Hematoxylin was used as a counterstain in those sections labeled for S100. Embedding and staining of mouse brain tissue was done through Yale Pathology Tissue services.

### Histological quantification

Quantification was performed using three Gabbr2 cKO and three control animals. To determine the number of S100-positive neurons in the cortex, four different regions at the same magnification were imaged for each mouse, and S100-positive neurons were counted within the standardized fields using Image J software (version 1.54p; http://imagej.org). The total number of cells in each field was counted using the “analyze particles” feature of ImageJ. In brief, images were first adjusted to 8-bit, thresholds were adjusted to visually discern single cells, images were changed to binary, converted to mask, the watershed function was applied, and then the “analyze particle” function was used with the edges being excluded.

In addition, four defined fields of Luxol blue-stained cerebellum were examined for each mouse. Purkinje neurons were counted in each image, and after setting the scale, the length of the cerebellar surface was measured using the freehand line function, which was used to normalize the number of Purkinje neurons over the Purkinje layer of the cerebellum. Individual dendrite lengths were also measured using the freehand line function.

In the hippocampus, we measured the width of the granular layer using the line function and determined the number of granular neurons in the layer. As before, we performed these measurements of the granular layer from 4 images per mouse. The number of granular neurons, vacuolated neurons in the granular layer, total number of pyramidal neurons, and the number of shrunken pyramidal neurons were all manually counted in standardized fields from the region of the dentate gyrus and CA3 region of the hippocampus. Vacuolated neurons were neurons with apparent cytoplasmic vacuoles, which have been described in aging, traumatized, or dying neurons [21]. Shrunken pyramidal neurons were identified by a clearing around the soma within the hippocampus.

### Motor agility

To examine motor changes in cKO compared to control mice, we assessed rotarod performance in mice that were 9–13 weeks old. Mice underwent 5 training sessions per day on the accelerating rotarod (AccuScan Instruments) over two separate days preceding the experiment. The initial speed of the rotarod was 0 rpm with a constant acceleration of 0.2 rpm/sec. Each training interval was for up to 300 sec and the amount of time that the mice remained on the rod was measured and recorded. During the test day, we recorded the length of time each mouse remained on the rod immediately before (time 0) and 1, 2, and 4 hours after the administration of L-baclofen (12.5 mg/kg) or vehicle (saline). This dose of baclofen has been reported to maximally affect rotarod performance in previous studies [6,22].

### Behavioral experiments

We examined hyperactivity and unsupported rearing in cKO vs control mice. Male mice between 6 and 8 weeks of age were used for these experiments. Before behavioral testing, the investigator individually handled mice (3 times over 72 hours before the test day) to decrease anxiety. Next, mice were placed in a new, empty home cage where unsupported rearing and locomotor activity were monitored and video recorded for 10-minute sessions, with the last 5 minutes manually scored for these behaviors. Unsupported rearing was defined as rearing without any contact with the walls of the test cage. The investigator was blinded as to the genotypes of the mice during scoring.

### Statistical analysis

Data are presented as mean± standard error (SE). Comparisons between two groups were conducted using Student’s unpaired two-tailed t-tests. Where appropriate, two-way ANOVA with Sidak multiple comparison tests were used. In experiments where multiple measurements were made on the same mouse, data was analyzed using a mixed effects model to effectively account for within subject correlation of repeated measures and between subject variability. All analyses were performed using Prism 10 (GraphPad Software, La Jolla, CA).

## Results

### Insertion of LoxP sites and reduction in GABBR2 expression

Using CRISPR we inserted loxP sites into 5’ and 3’ sites flanking exon 10 of the Gabbr2 gene, which encodes the first transmembrane domain of the receptor (Fig 1A). We targeted this exon for several reasons. First, it was predicted to result in the loss of the first transmembrane domain. Second, the excision of this portion of DNA was predicted to result in a frameshift and mistranslation of all downstream exons when the primary transcript was spliced. Both characteristics were predicted to result in a nonfunctional protein that would be degraded. Finally, targeting this relatively small exon allowed both flanking loxP sites to be targeted with one oligomer, allowing for more efficient editing. Primers were designed to detect wild-type and loxP sites at the 5’ and 3’ end of exon 10 to detect the appropriately floxed alleles (Fig 1B). Using these primers, we identified 5 potential founder lines in a FVB background and 2 founder lines in a C57Bl/6 background that contained both loxP sites (Fig 1C). Lines 19 and 33 in an FVB background were used for the following experiments, both of which produced offspring genotypes in the expected Mendelian fashion, (referred to as Gabbr2^lox/lox^ mice) (Fig 1D).

**Fig 2:**
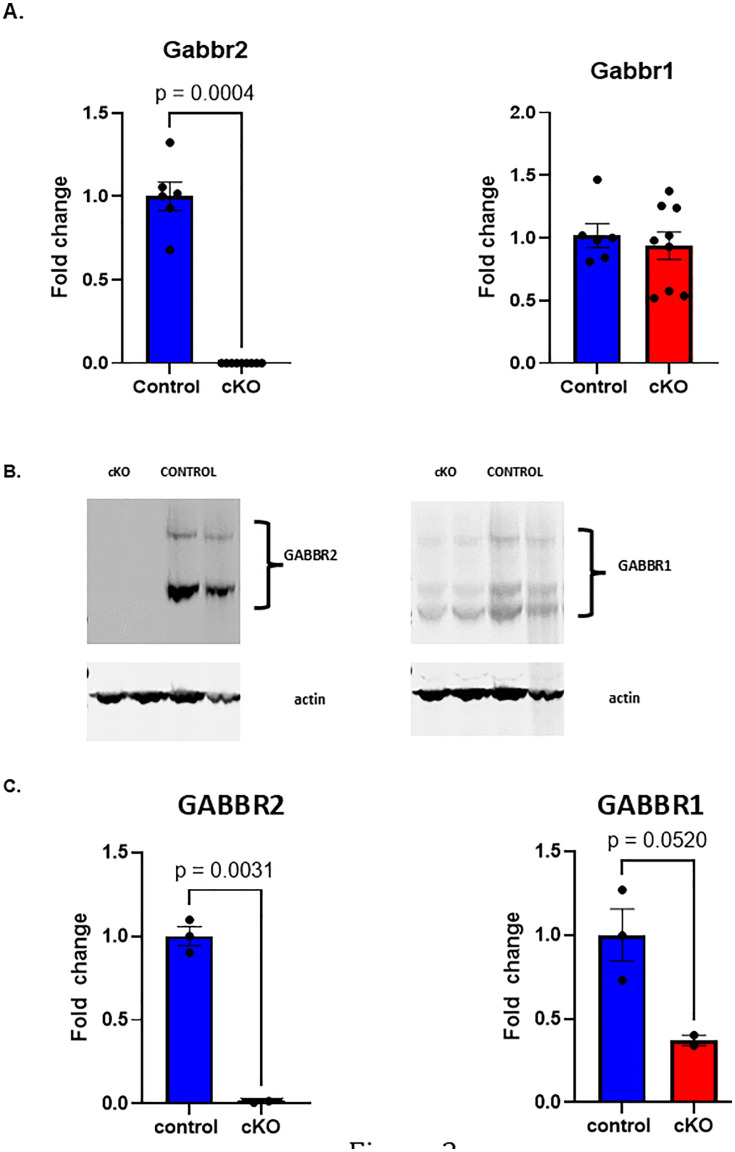
Expression of GABBR2 and GABBR1 in the brains of control and Gabbr2 cKO mice at 21-days old. A) QPCR to assess *Gabbr1* and *Gabbr2* mRNA levels in whole-brain RNA. The specific transcript is shown on the top of each graph. Bars represent the mean± SEM, n = 6 control (blue bars) and n = 9 Gabbr2 cKO (red bars) B) Typical immunoblots showing levels of GABBR2 and GABBR1 in whole-brain extracts. C) Quantification of immunoblots for GABBR2 and GABBR protein levels relative to the control. All bars represent the mean ± SEM. N = 2 control and n = 3 Gabbr2 cKO. p-values are presented on the graph.

Gabbr2^lox/lox^ mice were bred to B6.FVB-*Tmem163*^*Tg(ACTB-cre)2Mrt*^/EmsJ (Actin-cre) mice to generate Gabbr2 cKO mice with widespread loss of GABBR2 expression. In order to verify the loss of GABBR2, we examined *Gabbr2* mRNA levels in whole brains from 21-day-old mice. *Gabbr2* mRNA expression was greatly reduced in the Gabbr2 cKO mice as compared to Gabbr2^lox/lox^ (control) mice, lacking Cre expression (Fig 2A). Loss of *Gabbr2* mRNA expression did not affect Gabbr1 mRNA (Fig 2A). We also assessed GABBR2 protein expression by performing immunoblots of whole brain extracts. As shown in Fig 2B and 2C, and S2 Fig, no GABBR2 protein was detected in extracts of whole brains harvested from cKO mice, although it was easily detected in brain extracts from control mice. However, loss of GABBR2 protein did reduce GABBR1protein levels (Fig 2B and 2C, and S1A and S1B Fig). These results demonstrate the effective elimination of GABBR2 expression when Gabbr2^lox/lox^ mice are bred with Cre recombinase-expressing mice.

### Histological changes in the brain due to the loss of GABBR2

**Fig 3:**
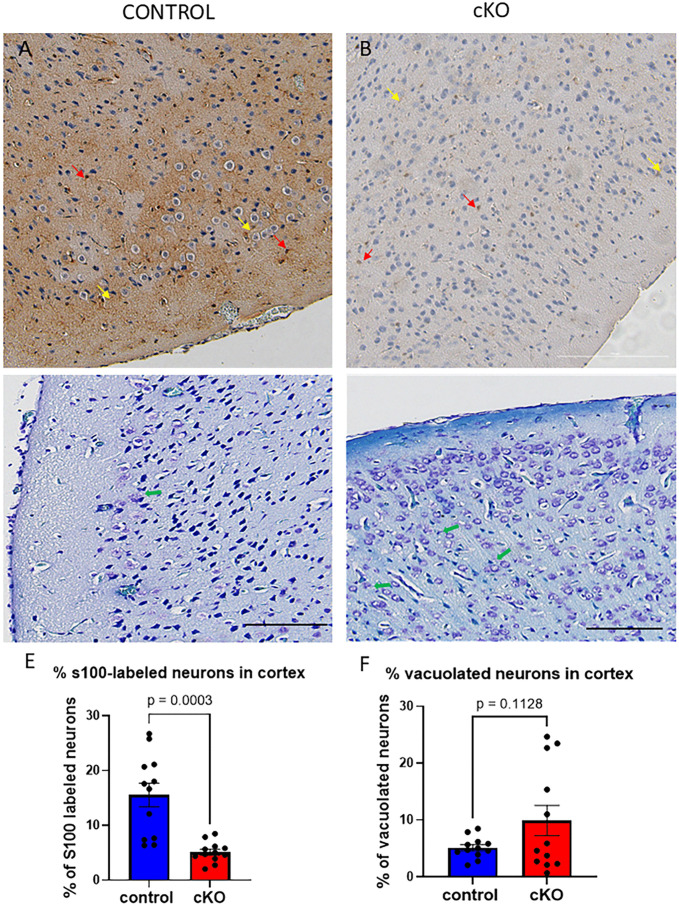
S100 immunostaining and Luxol Blue staining of cerebral cortex. A & B) S100 immunostaining of cerebral cortex from control mice (A) and Gabbr2 cKO mice (B) and counterstained with hematoxylin, which stains the nuclei. There was a significant reduction of S100 staining overall and fewer S100-labeled neurons in the Gabbr2 cKO cortex as compared to the control cortex. Yellow arrows point to neuronal dendrites. Red arrows point to S100-labeled neuronal bodies. C&D) Luxol Blue staining of cerebral cortex from control mice (C) and Gabbr2 cKO mice (D). There appeared to be more vacuolated neurons and cell debris in the cortex of cKO mice (D) as compared to controls (C) (green arrows). E&F) Quantification of S100-labelled neurons (E) and vacuolated cells (F) as a percentage of total cell numbers. There was a significant decrease in the percentage of neurons that stained for S100 but only a trend for an increase in vacuolated cells (F). Mice were 8 to 10 weeks of age. Three mice from each genotype were examined for quantification. Scale bar = 200 microns.

GABBR2 is expressed throughout the brain, including the cerebral cortex, cerebellum, Purkinje neurons, hippocampus, CA3 neurons, thalamic nuclei, medial habenula, and astrocytes [23–27]. S100 proteins are expressed diffusely in glial cells, astrocytes and neurons throughout the brain [28,29]. In the Gabbr2 cKO cortex, there was a generalized decrease in diffuse S100 staining and fewer distinct S100-positive cells when compared to control mice (Fig 3A versus 3B, red arrows, S3 Fig). In addition, there were fewer S100-positive neurons in the Gabbr2 cKO cortex (Fig 3E, S3 Fig). There was also an increase in vacuolated neuronal bodies and cell debris was evident in Luxol blue stained sections (Fig 3C, D, green arrows, Fig 3F, S3 Fig), suggesting potential neuronal damage.

**Fig 4:**
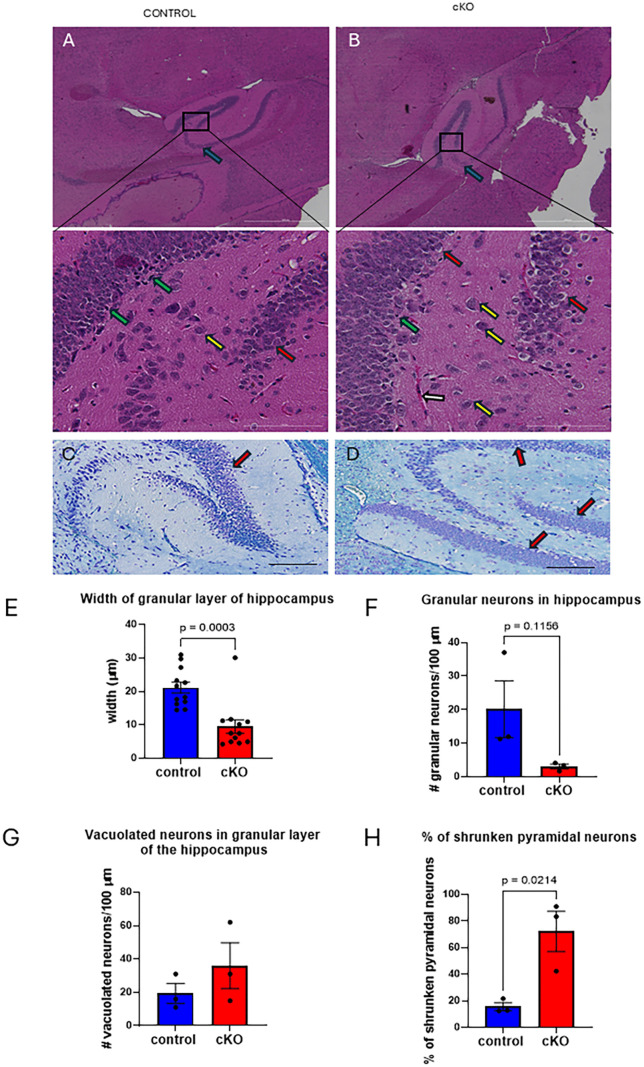
Hippocampal histology in Gabbr2 cKO mice. A&B) Shown is hematoxylin and eosin staining of the hippocampus focusing on the polymorphic layer of the dentate gyrus, where CA3 neurons are identified with blue arrows. In the inset at higher magnification, green arrows identify the dense immature granular neurons. The yellow arrows identify pyramidal neurons of the polymorphic layer, and red arrows identify vacuolated granular cells. A congested capillary is present in the Gabbr2 cKO hippocampus (inset from B, black arrow). C&D) Luxol Blue staining of hippocampus from control (C) and Gabbr2 cKO mice (D). Red arrows point to vacuolated granular cells. E) Quantification of the granular layer width in the hippocampus. F) Quantification of granular neurons per 100 microns length of granular layer. G) Quantification of the number of vacuolated neurons in the hippocampus per 100 microns length of hippocampus. H) Percentage of shrunken pyramidal neurons. Mice were 8 to 10 weeks of age. Bars represent the mean ± SEM. Quantification was performed on three mice per genotype. Blue bars are controls and red bars are Gabbr2 cKO mice. Scale bar = 1000 microns in A and B, 200 microns in the insets, and 100 microns in C and D. Two-sided t-tests were used to determine significance, except in E, where a mixed effects mixed model was used to determine significance.

Changes were also evident in the dentate gyrus and CA3 region of the hippocampus of Gabbr2 cKO mice. Staining of the CA3 region appeared reduced in Gabbr2 cKO mice (Fig 4A versus 4B, blue arrows). At higher magnification, one could appreciate a reduction in the number and layers of dense immature granular cells (Fig 4A versus 4B, green arrows; dotted borders in S4 Fig) as well as an increase in vacuolated cytoplasm in granular cells (Fig 4A versus 4B, and Fig 4C versus 4D, red arrows). The reductions in the width of the granular layer and the number of granular neurons in Gabbr2 cKO mice were quantified in (Fig 4E and 4F). There was no significant difference in vacuolated neurons in the granular layer (Fig 4G), but there were significantly more shrunken pyramidal cells in the polymorphic cell layer (Fig 4A and 4B, yellow arrows, Fig 4H) of the hippocampus in Gabbr2 cKO mice.

**Fig 5:**
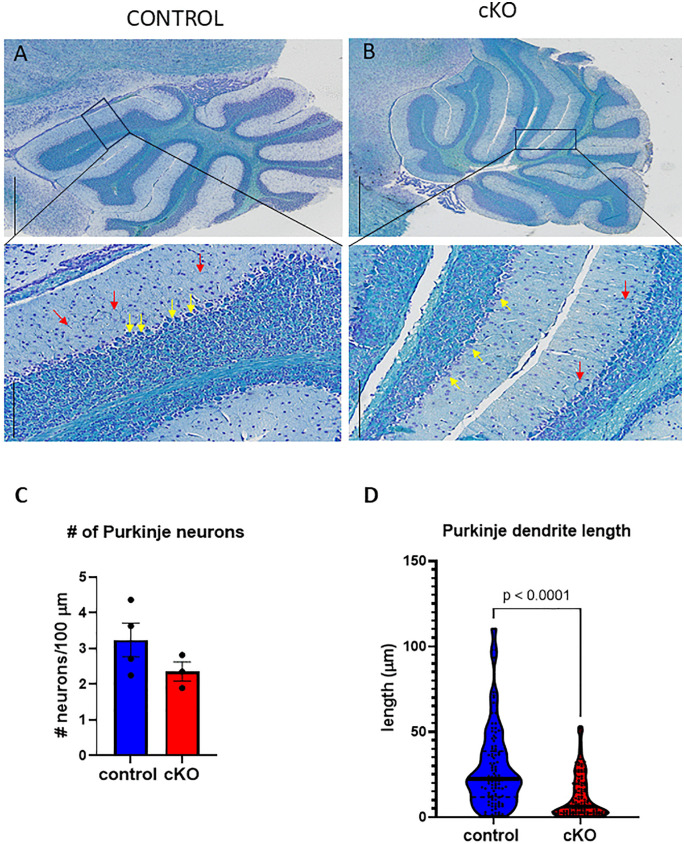
Cerebellum of Gabbr2 cKO and control mice. A&B) Luxol blue staining of representative cerebellar sections of control (A) and Gabbr2 cKO (B) mice. Luxol Blue staining intensity appeared to be reduced in the cerebellum of the Gabbr2 cKO mice compared to the control mice. Purkinje neurons were present in similar numbers between cKO and control mice. (yellow arrows). However, there were shorter, fewer and less complex dendritic extensions from the Purkinje neurons (red arrows) in the cKO cerebellum (inset from B compared with inset from A). C) Quantification of Purkinje neurons per 100 microns of Purkinje layer length. D. Violin plots showing the quantification of dendrite length in Purkinje neurons in Gabbr2 cKO versus control mice. Mice were 8 to 10 weeks of age. Quantification was performed on three mice per genotype. Purkinje neuron dendrite analysis was based on 124 cKO and 107control Purkinje neurons. Two-sided T-tests were used to determine significant differences, except for in D), where a mixed effect model was applied. A and B Scale bar is 1000 microns. Insets from A and B scale bar are 200 microns.

Finally, the cerebellum of Gabbr2 cKO mice demonstrated alterations in the organization of the Purkinje cell layer (Fig 5A versus 5B, yellow arrows). Although there were no differences in the total number of Purkinje neurons (Fig 5C), there was a reduction in the complexity of the dendritic branching pattern in the cKO cerebellum. Specifically, we observed fewer dendritic projections penetrating the molecular layer (Fig 5A and Fig 5B, red arrows), which is quantified in Fig 5D.

### Loss of GABBR2 alters behavior and motor skills

**Fig 6:**
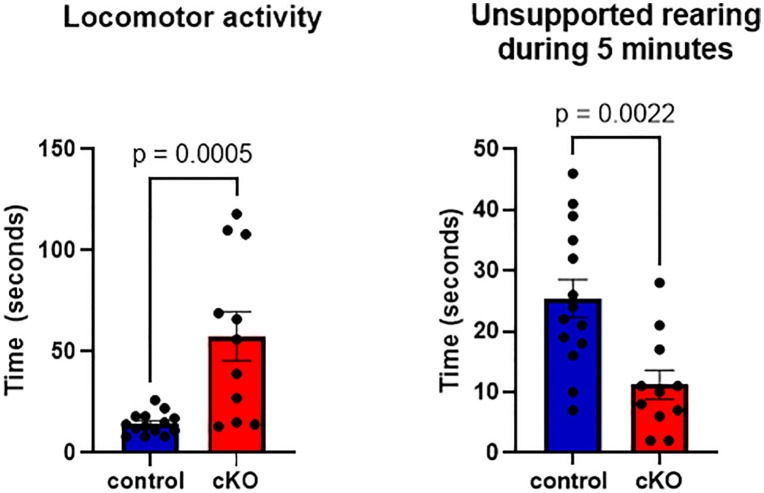
Locomotor activity and unsupported rearing in Gabbr2 cKO versus control mice. Increased locomotor activity and decreased unsupported rearing are seen in Gabbr2 cKO mice (red bars) vs. controls (blue bars). Bars represent the mean ± SEM. n = 14 for control mice, n = 11 for Gabbr2 cKO mice. Mice were 9-13 weeks of age.

Previous reports on the global Gabbr2 KO mice described hyperlocomotion, elevated anxiety-related behaviors, and spontaneous seizure activity [6,30]. Therefore, we examined these activities in Gabbr2 cKO mice to determine whether they mimicked the phenotype of global Gabbr2 KO Mice. Gabbr2 cKO mice demonstrated a greater than 3-fold increase in locomotor activity compared to control mice (Fig 6). There was a significant reduction in unsupported rearing behavior in Gabbr2 cKO mice as compared to controls (Fig 6). This decrease in exploratory behavior is likely indicative of increased levels of stress but can also be seen in the setting of neurodegenerative disorders [31–33].

**Fig 7:**
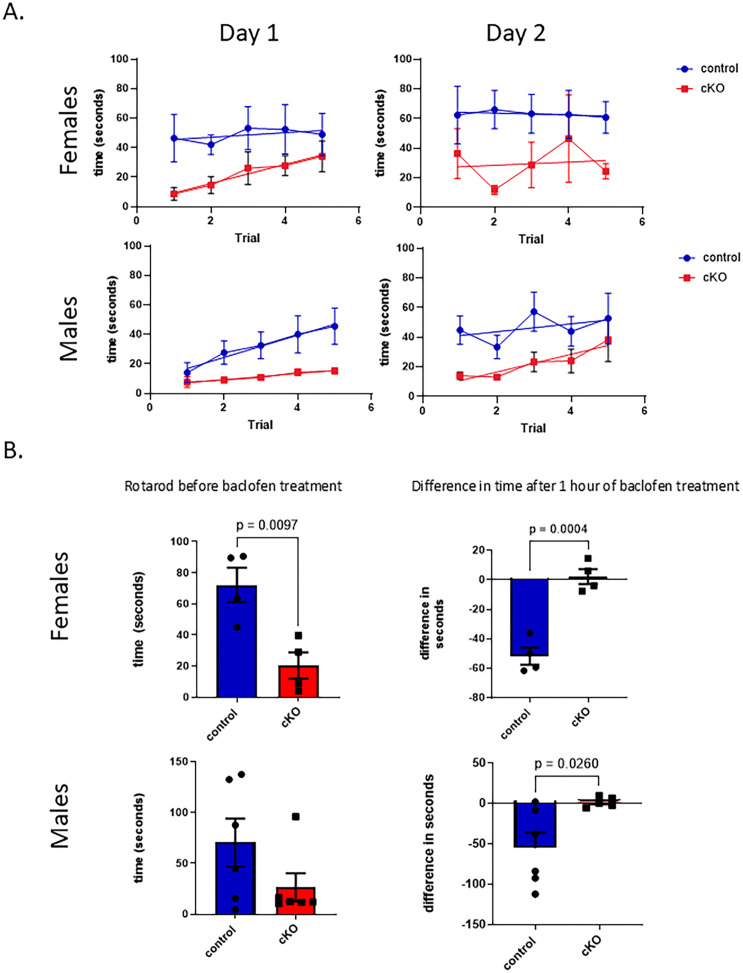
Rotarod experiments in control and Gabbr2 cKO mice. A). Training periods over 2 separate days showing times on rotarod for control (blue) and cKO mice (red). Points represent the mean ± SEM dwelling times over 5 trials on each of 2 days. Note that Gabbr2 cKO mice stay on the rod for shorter periods at baseline, although they do improve with training. B). Rotarod experiments in female and male control and Gabbr2 cKO mice before and after administration of baclofen, a GABBR2 agonist. Mice were 9 to 13 weeks of age. N = 6 for males for each genotype and N = 4 for females for each genotype. Bar graphs on the left demonstrate the absolute dwell times on the rotarod. Bar graphs on the right show the change in dwell time relative to baseline in response to baclofen administration. Bars represent mean ± SEM. Two-sided t-tests were used to determine statistical differences.

Baclofen is an agonist for GABA-B receptors and acts as a muscle relaxant [34,35]. Global Gabbr2 knockout mice were previously shown to be refractory to baclofen as measured by changes in rotarod performance [6]. Therefore, we assessed rotarod performance and responses to baclofen in Gabbr2 cKO mice and controls. During the rotarod training period preceding baclofen administration, it was clear that Gabbr2 cKO mice of both sexes had a baseline decrease in their ability to remain on the rotarod (Fig 7A). Therefore, we expressed the response to baclofen as the change from baseline (Fig 7B). Control mice of both sexes had a clear decrease in rotarod performance after baclofen treatment. However, despite the reduced performance at baseline, Gabbr2 cKO mice showed no additional decline in performance after administration of baclofen (Fig 7B, S5 Fig).

### Loss of GABBR2 results in seizures and premature death

**Fig 8:**
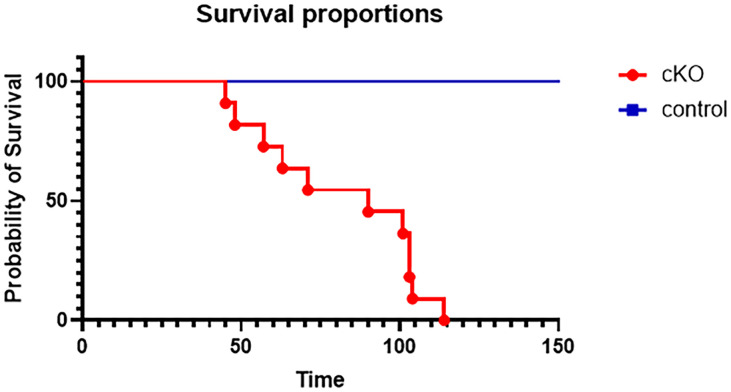
Kaplan-Myer plot showing survival curves of control and Gabbr2 cKO mice. No control mice died over 150 days of observation. 100% of Gabbr2 cKO mice died by 115 days. n = 11 in each group. Gehan-Breslow-Wilcoxon test (p < 0.0001).

We did not detect obvious spontaneous seizure activity while Gabbr2 cKO mice were being monitored for locomotor activity. However, these mice had frequent seizures when subjected to stressful stimuli, such as being handled or placed on the rotarod (S1 Video). In addition, we noted an increase in premature mortality in Gabbr2 cKO mice. As shown in Fig 8, 100% of Gabbr2 cKO mice died by 115 days of age while no control mice died during the same period.

## Discussion

We generated floxed Gabbr2 mice, that when crossed with Cre-expressing mice can be used to target elimination of GABBR2 expression in different cell types. In this report, we crossed floxed Gabbr2 mice with Actin-Cre mice to produce widespread knockout of the *Gabbr2* gene. *Gabbr2* mRNA expression was greatly reduced in the whole brain and GABBR2 protein was not detected by immunoblot, documenting efficient disruption of the *Gabbr2* gene and validating these mice as useful models for targeted disruption of the Gabbr2 gene.

The phenotype of Actin-Cre Gabbr2 cKO mice was similar to the global Gabbr2 KO mouse [6]. These mice demonstrated increased locomotor activity but a decrease in unsupported rearing behavior as well as impaired motor coordination and balance and seizures in response to being handled. These neuro-behavioral changes were accompanied by changes in brain histology and were associated with a reduced lifespan. These results recapitulate prior studies, speaking to the importance of GABBR2 for overall brain health and, perhaps, whole-body physiology as well.

We found that the loss of GABBR2 led to histological changes in different areas of the brain. In the cortex, GABBR2 is expressed in many different neurons including excitatory and inhibitory GABAergic interneurons [36–38]. Loss of GABBR2 would be expected to impair slow inhibitory Gaba signaling to the interneurons connecting different regions of the cortex, perhaps resulting in a progressive decline in interneuron function. Loss of inhibitory interneuron signaling may also result in changes in cell viability as reflected here by a reduction of cortical thickness, a decrease in S100 staining, reductions in dendritic extensions and the presence of vacuolated neurons and cellular debris. Functionally, such a decline in neuronal populations might contribute to the loss of motor skills and cognitive neurological function which we observed in these mice (Fig 6 and Fig 7).

The hippocampus functions in memory and learning and GABBR2 expression typically occurs in the area near mossy fiber synapses that form the major excitatory input into the auto-associative network of pyramidal cells in the CA3 region [39]. The loss of inhibitory input by GABBR2 to CA3 neurons could produce excitotoxity resulting in an increase in the death of pyramidal neurons, as shown by the increase in shrunken pyramidal neurons, a marker of increased cell death (Fig 4H). Loss of neurons that govern lateral inhibition in the dentate gyrus can cause delamination of the granule cell layer and multilamellar discharges in response to cortical stimuli resulting in increased excitotoxicity [40]. In the Gabbr2 cKO mouse, progressive damage over time to the excitable neurons lacking GABBR2 input in the dentate gyrus of the hippocampus may contribute to their susceptibility to seizures and hyperexcitability (Fig 6). The histological phenotype of the Gabbr2 cKO hippocampus is reminiscent of patients with epilepsy with a loss of dentate hilar neurons that govern dentate granule cell excitability [41,42].

Cerebellar Purkinje neurons are known to express GABBR2 [43]. Purkinje neurons project to the intermediate discharge layer and are the key efferent output of the cerebellum. The Gabbr2 cKO mice have Purkinje neurons (Fig 5, yellow arrows) with smaller dendritic arbors (Fig 5, red arrows, and Fig 5D) likely contributing to the abnormal rotarod performance (Fig 7). Purkinje dysfunction may also lead to fewer connections between the Purkinje neuron’s dendritic arbors and the interneurons in the molecular layer which may increase glutamatergic stimuli and neurotoxicity. Some seizure disorders cause increased Purkinje death by glutamate excitotoxicity [44].

In conclusion, the phenotype of Gabbr2 cKO mice was similar to the global Gabbr2 KO mouse [6]. Actin-Cre Gabbr2 cKO mice demonstrate histological changes in multiple areas of the brain in addition to changes in rearing behavior and premature death (Fig 6). Although our studies do not discriminate between whether these changes are the result of altered brain development or due to progressive excitotoxic neuronal damage, use of an inducible Cre transgene combined with the Gabbr2 ^lox/lox^ mice could address this question in the future. Gabbr2 floxed mice provide a new tool to target tissue-specific GABBR2 signaling through Cre-mediated recombination and to allow study of GABBR2 function in different cell types without the potentially confounding effects of neurological dysfunction caused by global loss of GABBR2.

## Supporting information

S1 Fig Immunoblot of brain lysates from Gabbr2 cKO mice and controls. A) Quantification of immunoblot for Gabbr1, normalized to actin staining. B) Immunoblot of Gabbr1 and actin with mouse anti Gabbr1 and mouse anti actin antibody.
(TIF)

S2 Fig Immunoblot of brain lysates from Gabbr2 cKO mice and controls. A) Same Immunoblot as S1 Fig but showing the Gabbr2 staining. B) Same immunoblot as A. showing actin staining. C) Quantification of Gabbr2 normalized to actin staining.
(TIF)

S3 Fig Quantification of S100 labeled and vacuolated neurons in the cortex. Graphs include the total number of S100 labeled neurons in 4 sections in 3 cKO and 3 control cortexes and the percentage of S100 labeled neurons over all neurons in the field. In addition, the total number of vacuolated neurons and the percentage of vacuolated neurons over all neurons in the field are graphed. A two-way t-test was used to determine significance. The numbers on the graph indicate p-value.
(TIF)

S4 Fig Hippocampal immature granular layer in Gabbr2 cKO mice. A&B) Shown is hematoxylin and eosin staining of the hippocampus focusing on the polymorphic layer of the dentate gyrus. In the inset at higher magnification, green arrows identify the dense immature granular neurons. Dotted white lines show the difference in width of the granular neuron layer of the hippocampus. Scale bar = 1000 microns in A and B, 200 microns in the insets.
(TIF)

S5 Fig Individual recordings of Gabbr2 cKO mice and controls on rotarod before and after administration of baclofen. A) Individual recordings of female Gabbr2 cKO and control mice. B) The means of recordings over time of female Gabbr2 cKO mice and controls. C) Male individual recordings of Gabbr2 cKO and control mice. D) The means of recording overtime. E. The mean weights of Gabbr2 cKO and control mice. N = 6 per genotype for males, and N = 4 per genotype for females. Some standard error bars are not seen because they are small and hidden by the points on the graph.
(TIF)

S1 Video Example of Gabbr2 cKO mouse having a seizure. (MP4)
